# Evaluation of *Entamoeba histolytica* recombinant phosphoglucomutase protein for serodiagnosis of amoebic liver abscess

**DOI:** 10.1186/1471-2334-13-144

**Published:** 2013-03-21

**Authors:** Tan Zi Ning, Wong Weng Kin, Rahmah Noordin, See Too Wei Cun, Foo Phiaw Chong, Zeehaida Mohamed, Alfonso Olivos-Garcia, Lim Boon Huat

**Affiliations:** 1 School of Health Sciences, Universiti Sains Malaysia, Kubang Kerian, Kelantan, 16150, Malaysia; 2 Institute for Research in Molecular Medicine, Universiti Sains Malaysia, Penang, 11800, Malaysia; 3 Department of Medical Microbiology and Parasitology, School of Medical Sciences, Universiti Sains Malaysia, Kubang Kerian, Kelantan, 16150, Malaysia; 4 Departamento de Medicina Experimental, Facultad de Medicina, Universidad Nacional Autónoma de México, México D.F, 04510, México

**Keywords:** *Entamoeba histolytica*, Amoebic liver abscess, 2-DE, Western blot, Phosphoglucomutase, Diagnosis, rPGM-ELISA

## Abstract

**Background:**

Amoebic liver abscess (ALA) is the most frequent clinical presentation of extra-intestinal amoebiasis. The diagnosis of ALA is typically based on the developing clinical symptoms, characteristic changes on radiological imaging and serology. Numerous serological tests have been introduced for the diagnosis of ALA, either detecting circulating amoebic antigens or antibodies. However those tests show some pitfalls in their efficacy and/or the preparation of the tests are costly and tedious. The commercial IHA kit that used crude antigen was reported to be useful in diagnosis of ALA, however high antibody background in endemic areas may cause problems in its interpretation. Thus, discovery of well-defined antigen(s) is urgently needed to improve the weaknesses of current serodiagnostic tests.

**Methods:**

Crude antigen of *E. histolytica* was analysed by 2-DE and Western blot to identify a protein of diagnostic potential for ALA. The corresponding gene of the antigenic protein was then cloned, expressed and the purified recombinant protein was subsequently evaluated for serodiagnosis of ALA in an indirect ELISA format.

**Results:**

Analysis of crude antigen showed that phosphoglucomutase (PGM) has the diagnostic potential. Recombinant PGM (rPGM) showed 79.17% (19/24) sensitivity and 86.67% (195/225) specificity in diagnosis of ALA based on the COV of mean +1SD. There was no significant difference between rPGM-ELISA and IHA diagnostic kit in the diagnosis of ALA in terms of sensitivity and specificity at p-value < 0.05.

**Conclusion:**

In conclusion, rPGM-ELISA is found to be useful for serodiagnosis of ALA. Future studies will determine whether rPGM-ELISA also detects antibodies produced in amoebic dysentery and asymptomatic cases.

## Background

Liver abscess is commonly detected by radiological imaging technique, but it cannot differentiate between amoebic and pyogenic liver abscesses. Differential diagnosis depends on clinical grounds and amoebic serology [1]. The serological tests revealed varied sensitivity levels, depending on the presence or absence of invasive disease and the types of invasive disease. Interestingly, more than 90% of patients with amoebic liver abscess (ALA) developed detectable anti-amoebic antibodies at 7 – 10 days after onset of symptoms. This augurs well for the development of serodiagnosis against the infection [2-4].

Numerous studies indicated high sensitivity and specificity of crude amoebic antigen *e.g.* crude soluble antigen and excretory-secretory antigens in capturing amoebic antibodies for diagnosis of ALA [5-8]. However, the pitfalls of crude antigen are the need to maintain *E. histolytica* cultures and the mass production of the antigen, which are costly and tedious. Occasionally, this cocktail antigen preparation reveals false positivity [9]. IHA Cellognost® Amoebiasis Kit (Dade Behring Marburg GmbH, Germany) that uses crude antigen was reported to be useful in diagnosis of ALA, yet due to the high background in endemic areas, the diagnostic validity varied between 70% and 90% among different geographical locations [10-12]. Hence, a standardised serological test based on well-defined antigen(s) is needed to overcome the weaknesses.

Recombinant DNA technology offers large-scale production of defined antigens through prokaryotic expression system. Serodiagnosis of invasive amoebiasis using defined recombinant *E. histolytica* antigens of serine-rich protein (SREHP), Gal/GalNAc-specific lectin and 29 kDa cysteine-rich surface protein has been reported since 1990s [13]. The recombinant protein of SREHP showed sensitivity of 79% and specificity of 87% in diagnosis of ALA, yet its overall diagnostic validity was lower than the conventional tests that utilized crude antigen [14]. Even though both recombinant Gal/GalNAc-specific lectin and 29 kDa cysteine-rich surface proteins showed high validity of ≥ 90% sensitivity and specificity in serodiagnosis of amoebic dysentery and asymptomatic amoebiasis, they were not specific in the diagnosis of ALA [15,16].

Therefore, this study attempted to identify and analyse potential new antigenic protein(s) from crude antigen of *E. histolytica* using human ALA serum samples. The corresponding gene of the antigenic protein(s) was then cloned and expressed, followed by the diagnostic potential evaluation of the purified recombinant protein in an indirect ELISA format.

## Methods

### Human serum

Positive serum samples were obtained from 24 patients warded in Hospital Universiti Sains Malaysia (HUSM) who were diagnosed with ALA based on clinical symptoms; detectable abscess by ultrasound imaging; and positive serology with a commercial indirect haemagglutination kit, IHA (Cellognost® Amoebiasis Kit, Dade Behring Marburg GmbH, Germany). On the other hand, non-ALA serum samples with IHA seronegative were obtained from 33 patients, in which four serum samples were obtained from patients with pyogenic liver abscess (n = 4) whereas the others were obtained from patients infected with pathogens other than *E. histolytica*, which include enteropathogenic *Escherichia coli* (n = 1), *Shigella sonnei* Group D (n = 1), *Salmonella* spp. (n = 5), *Klebsiella pneumoniae* (n = 1), *Staphylococcus aureus* (n = 1), *Ascaris lumbricoides* (n = 1), *Escherichia coli* (n = 2), Coagulase-negative *Staphylococcus* (n = 1), *Stenotrophomonas maltophilia* (n = 1), *Toxoplasma gondii* (n = 9) and *Helicobacter pylori* (n = 6). In this study, all the six *H. pylori* sera were pooled into a single tube because the individual volumes were too little. In addition, a total of 197 blood donor serum samples were obtained from Department of Haematology & Blood Transfusion Unit at HUSM. All the human serum samples were collected and checked with IHA from the year 2008 onwards and kept at -20°C until used. This study was conducted in accordance with the requirement of Universiti Sains Malaysia Human Research Ethics Committee, USMKK/PPP/JEPeM (213.3. [10]).

### Maintenance of *E. histolytica* and preparation of crude soluble antigen (CSA)

*E. histolytica* HM-1:IMSS was axenically cultured and maintained in Diamond’s TYI-S-33 medium [17]. For CSA preparation, 10 × 10^6^ of *E. histolytica* trophozoites were mixed with 500 μL complete Lysis-M buffer supplemented with protease inhibitor cocktail (Roche, Germany) and 20 μL of 0.5 M iodoacetamide (Sigma, USA). The mixture was then sonicated (Branson, Mexico) at 10% amplitude for three cycles of 1 min sonication with 0.5 sec pulse-on and 0.5 sec pulse-off. The lysate was centrifuged at 10 000 × *g* for 10 min at 4°C to collect the CSA in the supernatant. Subsequently, the protein concentration was estimated using Bradford protein assay [18].

### Analysis of CSA antigenic protein profile *via* SDS-PAGE and Western blotting

Twenty micrograms of CSA per well was separated by 9% sodium dodecyl sulfate polyacrylamide gel electrophoresis (SDS-PAGE) using Bio-Rad Mini-Protean III Electrophoresis Cell (Bio-Rad, USA) at a constant current of 25 mA per gel for about 1 h and the separated proteins were electroblotted onto a 0.45 μm-pore-size nitrocellulose (NC) membrane *via* a semidry transblot apparatus (Bio-Rad, USA) at constant voltage of 15 V for 45 min. The NC membrane was blocked for 1 h at RT with 5% skim milk with 10 mM Tris-buffered saline (TBS), pH 7.2 as the diluent. Subsequently, the NC membrane was cut into multiple strips and incubated with human sera at a dilution of 1:200 in TBS containing 0.1% Tween 20 (TBST) for 2 h at RT. The excess serum was removed by washing (3 × 5 min) the strips with TBST. Next, the strips were incubated with a monoclonal mouse anti-human IgG conjugated with horseradish peroxidase (HRP) (Invitrogen, USA) at a dilution of 1:6000 for 1 h at RT. The unbound secondary antibodies were removed by washing (3 × 5 min) with 0.1% TBST. The Western blot signal was detected by enhanced chemiluminescence (ECL) reagent (Roche Diagnostics, Germany), captured using X-ray film (Kodak, USA). The sensitivity and specificity of the antigenic protein was then evaluated based on serum samples collected from ALA cases (n = 24), patients with infections other than amoebiasis (n = 33) and blood donors (n = 30).

### Identification of the antigenic protein *via* 2-DE and Western blotting

A 2-DE gel electrophoresis was performed to further separate the antigenic protein band. Proteins of CSA were first fractionated based on their isoelectric point (pI) using 3100 OFFGEL Fractionator (Agilent Technologies, Germany). The ImmobilineTM Dry Strip (GE Healthcare, UK) with linear pH range 3–10 with a 12-well setup was used. The fractionation of CSA was performed according to the manual provided by the manufacturer. In brief, the protein sample was prepared by gently mixing 1600 μL of OFFGEL stock solution (1.25X) with 400 μL of the CSA sample with total protein mass of 2 mg. A volume of 150 μL of OFFGEL sample was loaded into each well of IPG strip after gel rehydration with 40 μL of IPG strip rehydration buffer. Mineral oil used as cover fluid was pipetted onto the gel strip ends. The sample was focused with a maximum power of 200 mW, maximum current of 50 μA and typical voltages ranging from 500 to 4500 V until 50 kVh was reached in ~24 h. The typical starting voltages used were from 200 to 1500 V. A volume of 20 μL of the fractionated protein sample was mixed with 5 μL of 5X SDS sample buffer without boiling, and subsequently separated by SDS-PAGE. The separated proteins were then transferred onto NC membrane followed by Western blot analysis using pooled and individual ALA or IHA seronegative serum samples at 1:200 dilution.

### Mass spectrometry analysis and protein identification

The antigenic protein band was excised from the 2-D SDS-PAGE gel and sent to Australian Proteomic Service for peptide sequencing by mass-spectrometry. At the proteomic facility**,** protein sample was digested with trypsin and the peptides were extracted and analysed by electrospray ionisation mass spectrometry (ESI-TRAP) using the Ultimate 3000 nano HPLC system (Dionex) coupled to a 4000 Q TRAP mass spectrometer (Applied Biosystems). The mass-spectrometry analysis was performed with two different gel slice samples to ensure reproducibility.

### Cloning of selected gene

Based on the amino acid sequence of the ~70 kDa antigenic protein, the encoding gene was identified. PCR primers were then designed based on the protein coding sequence retrieved from the GenBank (accession number XM_643579) to amplify the gene of interest from *E. histolytica* genomic DNA isolated from 3 × 10^6^ of *E. histolytica* trophozoites using a QIAamp DNA mini kit (QIAGEN GmbH, Germany). The optimum annealing temperature was 52°C. For cloning into pET-14b vector, *Xho*I (CTCGAG) and *BamH*I (GGATCC) recognition sequences were added to the forward and reverse primers, respectively. The primers sequences used to generate a 1785 bp PCR product were 5^′^- CCG CTC GAG ATG GCA CTG AAT AAT TAT ATT AAG-3^′^ forward primer; and 5^′^- CGC GGA TCC TTA CTC AGC TTT TGG TGG-3^′^ reverse primer. The amplified product with the expected size was cut and extracted from the 1% agarose gel using QIAGEN gel extraction kit (QIAGEN GmbH, Germany). Before ligation, both the PCR amplified product and pET-14b vector were digested with *Xho*I and *BamH*I (Fermentas Life Sciences, USA). The digested products were then purified by PCR purification kit (QIAGEN GmbH, Germany).

Ligation was performed by mixing the digested plasmid and PCR amplified product in the ratio of 1:3 in a final volume of 10 μL containing 1X T4 DNA Ligase buffer, 5 U of T4 DNA Ligase and dH_2_O. The ligation mixture was incubated overnight at 4 °C. Next, *E. coli* XL 1-Blue competent cells were transformed with the ligation mixture by heat-shock method. Colony PCR using T7 promoter primer (5^′^-TAATACGACTCACTATAGG-3^′^) and T7 terminator primer (5^′^-GCTAGTTATTGCTCAGCGG-3) was performed to screen for positive transformants, and the positive clones were confirmed by DNA sequencing.

### Expression and purification of recombinant antigenic protein

Recombinant plasmid carrying the open reading frame for the antigenic protein was transformed into expression host (*E. coli* BL-21 AI strain). The bacteria was grown at 37°C until OD_600 nm_ reached 1.2 and protein expression was induced with 0.2% (w/v) L-arabinose at 22°C for overnight. The recombinant protein was purified using nickel-nitrilotriacetic acid (Ni-NTA) affinity purification method. In brief, 800 mL of culture were harvested by centrifugation at 1800 × *g*, 20 min, at 4°C. The supernatant was discarded and the cell pellet was mixed and resuspended in 10 mL of lysis buffer [50 mM Tris–HCl (pH 7.5), 300 mM NaCl, 10 mM Imidazole and 1% Triton X-100] supplemented with 7.8 μL of β-mercaptoethanol. The suspension was then sonicated and centrifuged at 1800 × *g*, 30 min, at 4°C. The supernatant was transferred into a clean 15 mL centrifuge tube. A volume of 100 μL of Ni-NTA agarose (QIAGEN, Germany) was mixed with the supernatant and the suspension was then rotated at 4°C for 2 h. After that, the suspension was centrifuged at 450 × *g*, 2 min, at 4°C. The supernatant was discarded and the Ni-NTA agarose was washed with 10 mL of ice-cooled His-Tag washing buffer [50 mM Tris–HCl (pH 7.5), 300 mM NaCl and 20 mM Imidazole]. The suspension was rotated at 4°C for 30 min. The supernatant was discarded and the washing step on Ni-NTA agarose was repeated for another 5 times. In the final washing step, the suspension was centrifuged at 450 × *g*, 2 min, at 4°C and the supernatant was discarded. The recombinant protein that bound to the Ni-NTA agarose was eluted by 120 μL of ice-cooled His-Tag elution buffer [50 mM Tris–HCl (pH 7.5), 300 mM NaCl and 200 mM Imidazole] and rotated at 4°C for 3 h. The suspension was then centrifuged at 450 × *g*, 5 min, at 4°C and the concentration of the supernatant (eluted protein) collected in a clean 1.5 mL micro-centrifuge tube was determined by Bradford protein assay.

### Development and evaluation of indirect recombinant antigen based ELISA

Indirect-ELISA was performed on the human serum samples using the purified recombinant protein based on modifications of method described by Reen [19]. The coating concentration of the antigen for each well of a microtiter plate was 1 μg/mL; and the primary and secondary antibody dilutions were 1:50 and 1:250, respectively. In brief, each well of a 96-well flat-bottom microtiter plate (NUNC, Denmark) was coated with 100 μL of antigen diluted in 0.1 M bicarbonate buffer (pH 9.6). The plate was then covered and incubated overnight in a humid box at 4°C. Prior to the blocking step, coated plate was equilibrated for 1 h at room temperature and then each well was rinsed (5 × 5 min) with 200 μL phosphate buffered saline added with 0.05% Tween-20 (PBST). The plate was blotted against a piece of paper towel after every round of rinsing. After 1 h in blocking reagent (Roche, Germany), each well was washed with 0.05% PBST (5 × 5 min), followed by another hour of incubation with 100 μL of diluted serum samples, performed in duplicate. After another round of washing, 100 μL of PBS diluted HRP-conjugated mouse monoclonal anti-human IgG (Invitrogen, USA) was added into the well and incubated for 1 h. Following a final round of washing, 100 μL of TMB substrate was added and the plate was incubated in the dark at room temperature for 15 min. The optical densities (OD) were finally read at 450 nm VersaMax™ Microplate Reader (Sunnyvale, USA). The and specificity of the recombinant antigen in an indirect ELISA format was then evaluated against the IHA kit using the different categories of serum samples collected. The COV for the indirect ELISA was set at either mean OD value plus one or two standard deviations (SD) from the OD values obtained from 113 IHA seronegative blood donor serum samples.

### Statistical analysis

Statistical analysis was performed with SPSS for Windows version 18.0. The association between the indirect ELISA and IHA in clinical diagnosis of ALA was analysed using chi-square test; if indicated Fisher’s exact test was used instead.

## Results

### IgG blots of CSA

**Figure 1:**
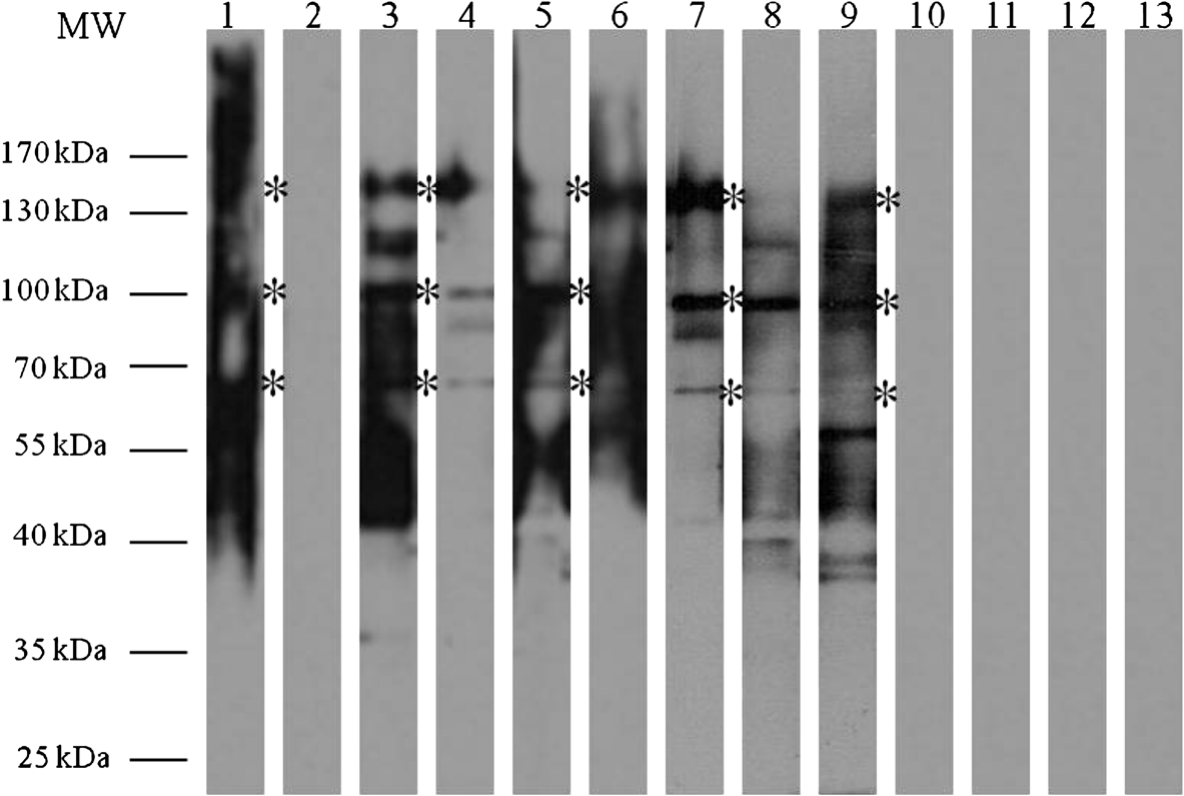
**Western blot profile of CSA probed with representative individual human serum.** Lane MW: Molecular weight standard (PageRulerTM Prestained Protein Ladder); Lane 1: Pooled human ALA serum; Lane 2: Pooled human IHA seronegative serum; Lanes 3 to 9: Individual human ALA serum; Lanes 10–12: Individual human IHA seronegative serum; Lane 13: TBS control. (*) indicates the estimated molecular weight of proteins (~170 kDa, ~100 kDa and ~70 kDa).

Immunoblotting results showed that there were three antigenic proteins *i.e.* ~170 kDa, ~100 kDa and ~70 kDa, recognized by individual human ALA serum samples (Figure 1). Based on the 24 human ALA serum samples, the sensitivities of these three antigenic proteins were 70.83%, 62.5% and 70.83%, respectively; while their specificities were 100%, 100% and 96.83%, respectively, when tested against a total of 63 negative control serum samples. The results on the sensitivity and specificity are summarised in Table 1.

**Table 1:** Sensitivity and specificity of each potential antigenic protein bands of CSA

**Molecular weight of antigenic protein band (kDa)**	**Clinically confirmed human ALA serum (n = 24)**	**Human IHA seronegative serum (n = 63)**	**Sensitivity (%)**	**Specificity (%)**
170	17	0	70.83	100
100	15	0	62.5	100
70	17	2	70.83	96.83

### 2-DE Western blot analysis and protein identification

**Figure 2:**
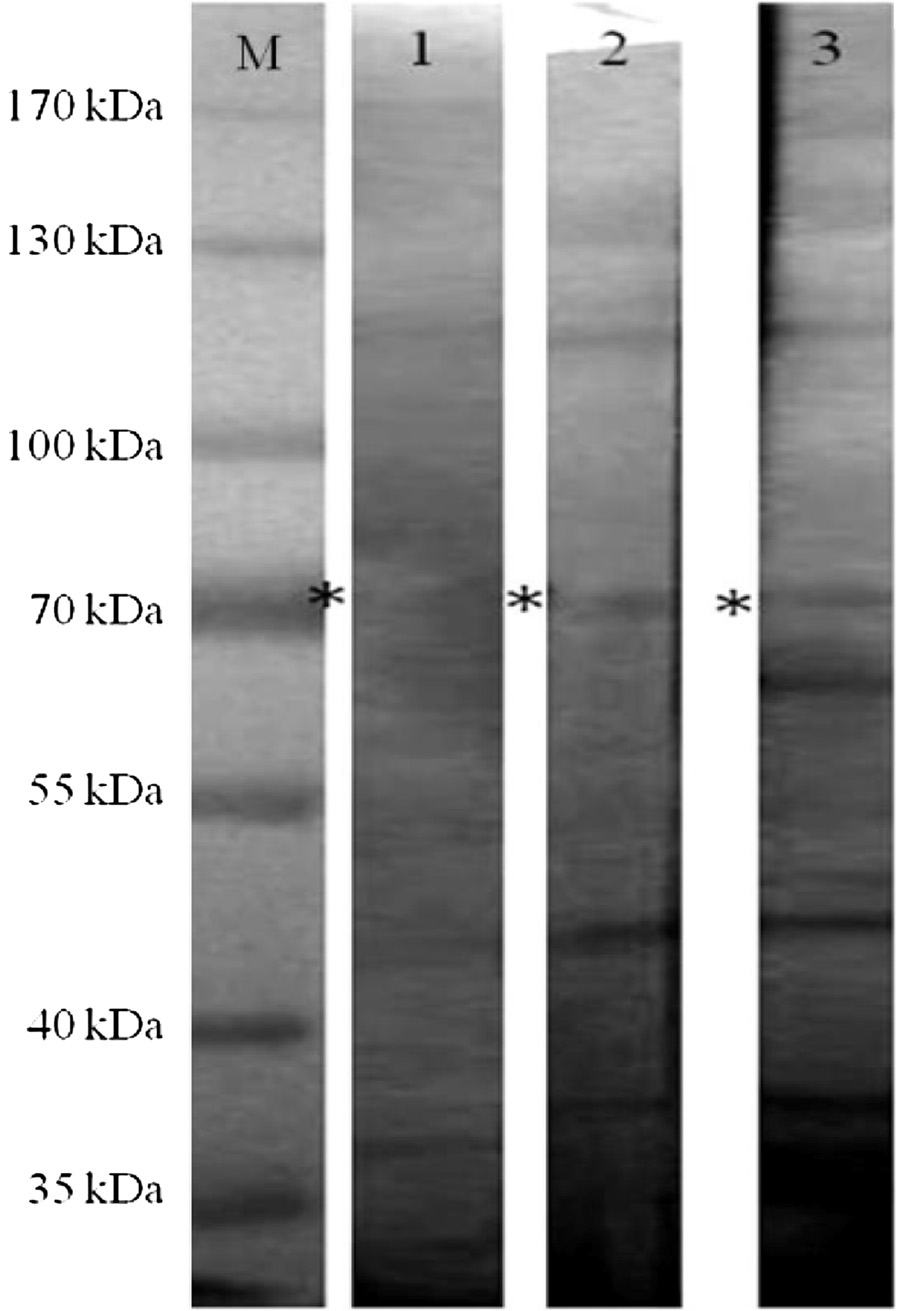
**Antigenic protein profile of fractionated CSA probed with human ALA serum.** Lane M: Molecular weight standard (PageRulerTM Prestained Protein Ladder); Lane 1: Pooled human ALA serum; Lanes 2–3: Individual human ALA serum; (*) indicates the targeted protein (~70 kDa).

Both the ~170 kDa and ~70 kDa proteins revealed almost similar sensitivity and specificity. Since the ~170 kDa was most probably the previously studied lectin antigen [16] and ~100 kDa showed lower sensitivity, hence here, only the ~70 kDa protein was further studied. In 2D-Western blotting analysis, the 70 kDa protein was presented in fraction 7 with pH between 6.5 and 7.08. Western blot in fraction 7 using two individual human ALA serum samples revealed positive reactivity against the targeted antigenic protein (Figure 2). Mass-spectrometry analysis identified the ~70 kDa antigenic protein as phosphoglucomutase (PGM) of *E. histolytica* (accession number C4M3Z6) with the protein score of 793 and 19 peptide sequences matched to the amino acid sequence. The score > 55 indicates identity or extensive homology at a significant level of p < 0.05. The total sequence coverage of the protein was reported as 25%.

### Expression and purification of the recombinant PGM (rPGM) protein

**Figure 3:**
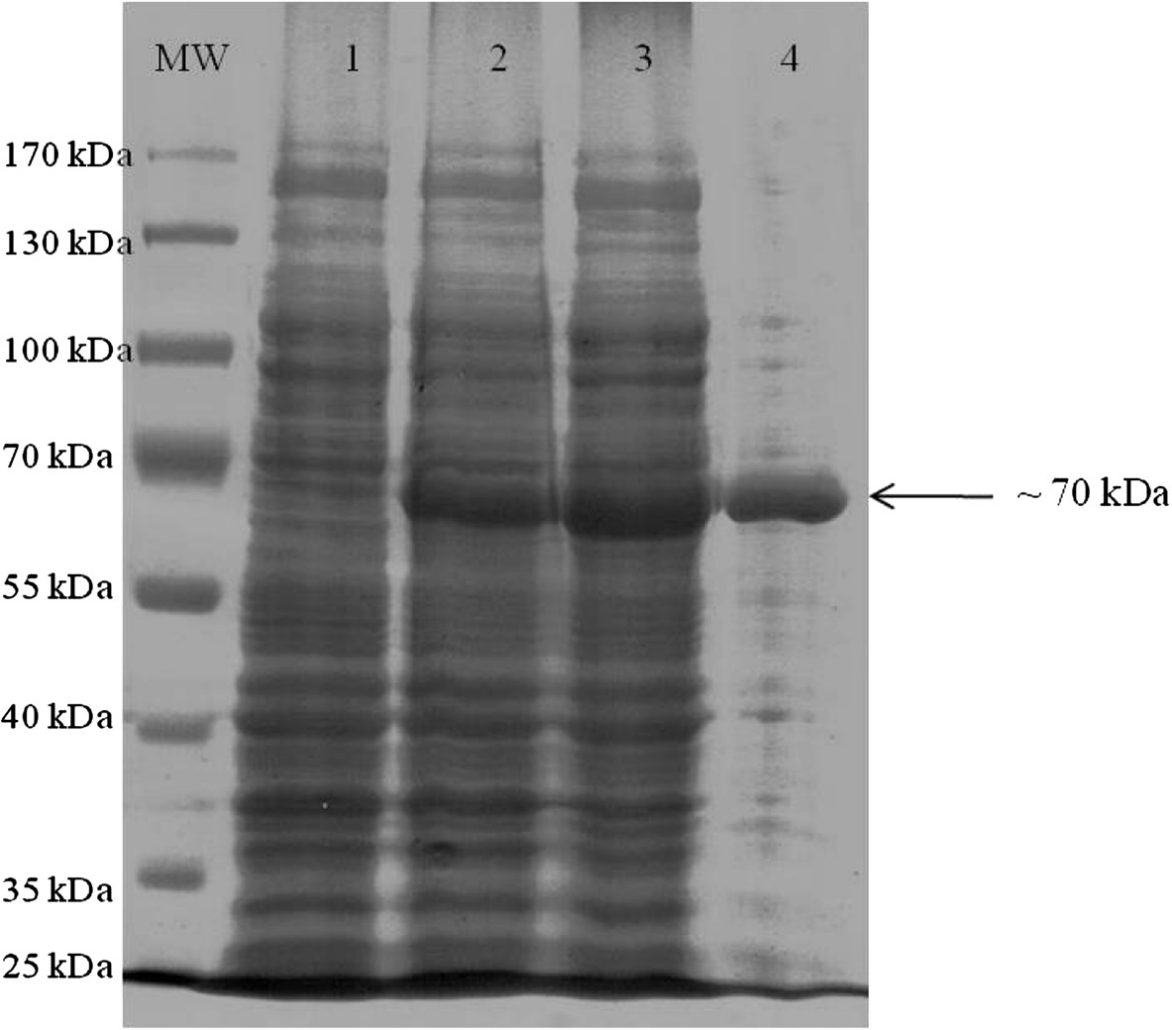
**Purified recombinant PGM protein.** Lane MW: PageRulerTM Prestained Protein Ladder; Lane 1: Non-induced cell pellet; Lane 2: Induced cell pellet; Lane 3: Induced cell supernatant; Lane 4: Purified protein; Arrow indicates the recombinant PGM protein (~70 kD).

The rPGM protein tagged with six consecutive histidine residues was purified using Ni-NTA affinity purification and was evaluated by 9% SDS-PAGE (Figure 3). The result showed that about 50% of the expressed PGM was present in the soluble fraction (lane 3). In lane 4, it revealed the soluble recombinant protein was successfully purified to apparent homogeneity.

### Efficacy of rPGM-ELISA

The COVs of rPGM-ELISA at mean OD value + 1SD and mean OD value + 2D were determined to be 0.25 and 0.32, respectively. The sensitivity was calculated based on results of 24 human ALA serum samples; while a total of 225 human sera from other infections and blood donors were used for specificity test. Based on the COV of mean + 1SD and mean + 2SD, the sensitivities of rPGM-ELISA were 79.17% (19/24) and 45.83% (11/24), respectively; while the specificities of the tests were 86.67% (195/225) and 93.78% (211/225), respectively (Table 2). Thus the COV based on mean + 1SD was selected over the COV of mean + 2SD as it showed better sensitivity and specificity in diagnosis of ALA. In comparison with IHA kit, statistical analysis showed that there was no significant difference between rPGM-ELISA and IHA in terms of sensitivity and specificity at p-value < 0.05 (Table 3).

**Table 2:** Number of serum sample at different COV settings from different serum test groups using rPGM-ELISA

**rPGM-ELISA (COV = mean + 1SD)**	**Number of Serum samples**		**Total**
	**< 0.25**	**≥ 0.25**	
Human ALA serum	5	19	24
Other infections human serum	21	7	28
Blood donors’ serum	174	23	197
Total	200	49	249
**rPGM-ELISA (COV = mean + 2SD)**	**< 0.32**	**≥ 0.32**	
Human ALA serum	13	11	24
Other infections human serum	26	2	28
Blood donors’ serum	185	12	197
Total	224	25	249

**Table 3:** Comparison between rPGM-ELISA and IHA in terms of sensitivity and specificity in diagnosis of ALA using statistical analysis

**Sensitivity**		**IHA**		**Total**	**Fisher’s Exact Test (p-value)**
		**.00**	**1.00**		
rPGM-ELISA	.00	0	5	5	0.620
	1.00	2	17	19	
Total		2	22	24	
**Specificity**		**IHA**		**Total**	**Fisher’s Exact Test (p-value)**
		**.00**	**1.00**		
rPGM-ELISA	.00	181	14	195	0.638
	1.00	28	2	30	
Total		209	16	225	

Note: (.00): Negative; (1.00): Positive.

## Discussion

Conventionally, diagnosis of ALA is confirmed by finding the *E. histolytica* trophozoites in liver pus aspirate obtained *via* ultrasound guided percutaneous aspiration biopsy, but the parasites are often absent as most of them are located at the margin on the peripheral of the abscess [20,21]. Therefore, serodiagnosis is widely adopted for diagnosis of ALA, detecting either amoebic antigens or antibodies from serum samples. Although numerous commercial antigen detection kits have been developed *i.e.* TechLab *E. histolytica* II ELISA (TechLab, Blacksburg, VA), *Entamoeba* CELISA-PATH (Cellabs Pty Ltd., Brookvale, Australia), Optimum S *Entamoeba histolytica* antigen ELISA (Merlin Diagnostika, Berheim-Hersel, Germany), Triage parasite panel BIOSATE (diagnostic, San Diego, CA), and ProSpecT *Entamoeba histolytica* microplate assay (REMEL Inc., Lenexa, KS), only TechLab *E. histolytica* II ELISA was evaluated for the detection of circulating antigen in ALA patient serum samples [22]. Haque *et al.*[23] reported that the kit detected Gal/GalNAc lectin in the ALA patient serum samples with a sensitivity of 96%. However, reports by Parija and Khairnar [24] and Zeehaida *et al.*[25] revealed lower sensitivities at 50% and 8.6%, respectively.

Instead of antigen detection, diagnosis of ALA can be performed by detecting circulating antibodies because hepatic amoebiasis raises a strong humoral response, especially immunoglobulin G [26]. Although a variety of serological assays was reported in diagnosis of ALA based on detecting circulating antibodies *i.e.* IHA, latex agglutination, indirect immunofluorescence, counter-immunoelectrophoresis, gel diffusion, complement fixation and ELISA; IHA and ELISA are still the preferred choices [22]. ELISA is commonly used as the routine diagnostic assay in diagnosis of ALA because it can be developed for in-house use based on different amoebic antigen preparations such as CSA, excretory-secretory antigens, plasma membrane antigens, purified antigenic proteins or recombinant proteins.

The CSA-based ELISA technique has either been used in routine diagnosis of ALA or field screening of amoebiasis [8,10,27,28]. In the diagnosis of ALA, CSA*-*based ELISA was reported to be 100% sensitive and > 90% specific [8,29]. Excretory-secretory antigens have been reported to be useful in diagnosis of ALA, which showed sensitivity of ~80% in Western blot analysis [7,30]. Another ELISA using amoebic plasma membrane antigen for diagnosis of ALA was reported to be 95% sensitive and 91% specific against anti-amoebic IgG; while detection of anti-amoebic IgM was 91% sensitive and 95% specific [31].

Nonetheless, crude preparations of amoebic antigens produced variable diagnostic efficacies, which may lead to false positive in the diagnosis of ALA. These antigen preparations require maintenance of *E. histolytica* cultures, which is costly and tedious. Moreover, the proteins are not well-defined; hence the interpretation of results may vary from batch to batch. In this study, analysis of the CSA protein profile showed that most of the antigenic proteins recognized by the human ALA serum antibodies ranged from 25 kDa to 170 kDa. Lower percentage (9%) polyacrylamide gel revealed that the ~170 kDa, ~100 kDa and ~70 kDa protein bands were well recognized by human ALA serum samples. Here, the ~70 kDa protein with 70.83% sensitivity and 96.83% specificity was selected for cloning and production of recombinant protein. The ~170 kDa protein also revealed almost similar sensitivity and specificity, but was not selected because it was most probably similar to the previously described lectin surface antigen of *E. histolytica*[16].

Prior to elucidating the identity of the ~70 kDa protein, it was further separated using 2-DE electrophoresis, a widely used technique in biomarker discoveries [32]. Proteins of CSA were fractionated *via* OFFGEL approach based on pI separation. This approach is advantageous when serum sample is limited. Multiple individual serum samples can be applied on the selected pI protein fraction containing the protein of interest in NC strip blot format, instead of using the whole gel NC membrane format in the conventional 2D-Western blot.

The ~70 kDa protein in this study was identified as *E. histolytica* PGM by mass-spectrometry analysis. This protein is reported to be involved in the glycolytic pathway of *E. histolytica*, which catalyses the inter-conversion of glucose 6-phosphate and glucose 1-phosphate. Previously, PGM was used as the gold standard for differentiation between *E. histolytica* and *E. dispar via* isoenzyme electrophoresis together with hexokinase [33]. Interestingly, there is no report on the value of this protein in diagnosis of invasive amoebiasis.

Well-defined antigens such as purified antigenic proteins and recombinant proteins of *E. histolytica* can overcome some of the setbacks posed by utilization of crude soluble preparations. For instance, purified lectin antigen and purified 29 kDa cysteine-rich surface protein in an ELISA format were reported to be useful for diagnosis of ALA [34,35]. According to Flores *et al.* (1993), the purified native 29 kDa cysteine-rich surface protein showed 79% sensitivity and 98% specificity in the diagnosis of ALA [34]. Several recombinant proteins have been produced such as SREHP, Gal/GalNAc-specific lectin, 29 kDa cysteine-rich surface protein and 40 kDa NADP^+^-dependent alcohol dehydrogenase (EhADH1) [13,36]. Recombinant proteins of SREHP, Gal/GalNAc-specific lectin and 29 kDa cysteine-rich surface protein have been evaluated for diagnosis of ALA. SREHP/MBP fusion protein, which was among the first recombinant proteins used in serodiagnosis of ALA was reported to have a sensitivity and specificity of 74% and 55%, respectively. Interestingly, its diagnostic validity increased to 79% and 87%, respectively, after the removal of its MBP component [14]. The validity is almost similar with rPGM protein in the current study, which is 79.17% sensitive and 86.67% specific. Recombinant full-length 170 kDa Gal/GalNAc-specific lectin protein has been produced and its validity in diagnosis of ALA was about 91% sensitivity [16]. Besides, several recombinant fragments of the Gal/GalNAc-specific lectin protein have also been investigated for the diagnosis of ALA. For example, recombinant protein of a truncated immunodominant domain of the Gal/GalNAc-specific lectin protein with 1.85 kbp cDNA insert showed more than 90% for both sensitivity and specificity [28]. Another study using recombinant Gal/GalNAc-specific lectin-derived protein (LC3) showed 100% sensitivity in diagnosis of ALA [37]. A study carried out by Tachibana *et al.*[29] reported that the 150 kDa recombinant intermediate subunit (IgI) of the Gal/GalNAc-specific lectin protein used in diagnosis of ALA was only 8.6% sensitive. A recombinant 29 kDa cysteine-rich surface protein was also developed and evaluated as its purified form showed moderate validity in diagnosis of ALA. The evaluation of this recombinant protein in the diagnosis of ALA showed increment of sensitivity (90%) compared to its purified native form protein (79%) [34].

Although several recombinant proteins have been evaluated, they were not specific for diagnosis of ALA because several studies have reported that those recombinant proteins were immunoreactive with serum samples for amoebic dysentery and asymptomatic cases [15,16,29,38,39]. In the current study, the novel rPGM protein showed diagnostic validity of ~80% sensitivity and ~90% specificity in diagnosis of ALA. The efficacy of rPGM-ELISA was almost comparable to previously reported recombinant proteins such as SREHP, Gal/GalNAc-specific lectin and 29 kDa cysteine-rich surface protein, which showed both sensitivity and specificity ranging from 80% to 100% [16,34,39].

## Conclusion

In conclusion, the novel rPGM protein used in an indirect ELISA format developed in this study could be useful for in-house routine diagnosis of ALA. Future studies will determine whether rPGM-ELISA also detects antibodies produced in amoebic dysentery and asymptomatic cases.

## Consent

Written informed consents were obtained from the patients for publication of this report.

## Competing interests

The authors hereby declare that they have no competing interests.

## Authors’ contributions

All authors contributed to this work. First author, TZN carried out the experiments and drafted the manuscript. All authors were involved in the manuscript preparation. All authors read and approved the final version of the manuscript.

## Pre-publication history

The pre-publication history for this paper can be accessed here:

http://www.biomedcentral.com/1471-2334/13/144/prepub
